# The influence of body mass index on physical activity engagement following anterior cruciate ligament reconstruction: A systematic literature review

**DOI:** 10.1016/j.heliyon.2023.e22994

**Published:** 2023-11-28

**Authors:** Srdjan Ninkovic, Marko Manojlovic, Roberto Roklicer, Antonino Bianco, Attilio Carraro, Radenko Matic, Tatjana Trivic, Patrik Drid

**Affiliations:** a Faculty of Medicine, University of Novi Sad, Novi Sad, Serbia; b Department of Orthopedic Surgery and Traumatology, Clinical Center of Vojvodina, Novi Sad, Serbia; c Faculty of Sport and Physical Education, University of Novi Sad, Novi Sad, Serbia; d Faculty of Education, Free University of Bozen-Bolzano, Brixen-Bressanone, Italy; e Sport and Exercise Sciences Research Unit, University of Palermo, Palermo, Italy

**Keywords:** Exercise, Knee injury, Obesity, Overweight, Surgery

## Abstract

**Background:**

The objective of this study was to summarize available literature that explored the impact of body mass index (BMI) on physical activity participation among individuals who were subjected to the anterior cruciate ligament reconstruction (ACLR).

**Methods:**

A total of three electronic databases, including Web of Science, Scopus, and PubMed, were comprehensively searched to identify relevant investigations. The following inclusion criteria were applied: (1) study design was observational; (2) participants underwent the ACLR; (3) BMI was estimated as a predictor variable; and (4) outcomes evaluated referred to physical activity. The risk of bias was assessed with the National Institutes of Health Quality Assessment Tool for Observational Cohort and Cross-Sectional Studies.

**Results:**

After a database search, 787 studies were found, and only 10 of them met each of the eligibility criteria and were included in the qualitative analysis. Regarding respondents' characteristics, 7171 individuals underwent ACLR, 4080 males and 3091 females, with a mean age of 25.5 years. Most importantly, the average BMI of the examined population was 24.9 kg/m^2^. In all studies, physical activity was evaluated subjectively using the Tegner activity scale and the Marx activity scale. The main findings unambiguously demonstrated that a negative relationship between BMI and physical activity engagement was observed. More specifically, there is convincing evidence that BMI over 25 kg/m^2^ harmfully affected subjectively assessed physical activity in individuals with a history of ACLR.

**Conclusion:**

The results obtained in the presented research indicated that increased values of BMI were a factor that correlated with reduced physical activity levels in the ACLR population. Hence, taking into account the clinical and health implications of reduced physical activity participation, stimulation of a healthy lifestyle, such as a combination of adequately designed physical exercise and nutrition, seems necessary for the analyzed population.

## 1 Introduction

Available scientific literature indicates that anterior cruciate ligament (ACL) rupture represents one of the most examined phenomena in the fields of sports science and medicine, as well as sports traumatology [1,2]. Moreover, ACL tear is considered the most frequent injury of the knee joint among athletes competing in contact sports [3]. Most importantly, following ACL rupture, ACL reconstruction (ACLR) was very efficient regarding the restoration of knee stability, improvements in joint kinematics, and a safe return to physical activity participation [4]. There is abundant evidence concerning the incidence of ACLR worldwide [2,[5], [6], [7]]. A total of 238,810 ACLRs have been reported over the last several decades among adolescent females in the United States [5]. It is also noteworthy to emphasize that the primary ACLR was far more prevalent than the revision of the ACLR in the Canadian population [2]. In terms of the incidence of the ACLR, the importance of gender and age was highlighted in the literature [6]. Specifically, the number of ACLRs was substantially higher in females compared to males. In addition, the authors reported that ACLR commonly occurs in participants younger than 20 years and older than 40 years.

Body mass index (BMI) is defined as the ratio of body weight expressed in kilograms to the squared values of body height expressed in meters [8]. According to the World Health Organization, four uniform categories of BMI have been established, including underweight, normal weight, overweight, and obesity [9]. The range of BMI between 15 and 19.9 kg/m^2^ indicated underweight, BMI between 20 and 24.9 kg/m^2^ referred to normal weight, the range of BMI between 25 and 29.9 kg/m^2^ indicated overweight, and BMI over 30 kg/m^2^ referred to the obesity [10]. BMI is widely used as a measure to diagnose obesity in the area of national and international public health [11]. In general, obesity has been linked with various health parameters [[12], [13], [14]]. Of note, a robust body of evidence highlighted a positive correlation between obesity and cardiovascular disease, type-2 diabetes, and osteoarthritis [12]. Furthermore, the presence of obesity negatively impacted health-related quality of life in middle-aged and older men and women [13,14]. At last, cognitive functions were considerably impaired in adults with a BMI of 30 kg/m^2^ or greater [15].

In individuals that underwent ACLR, physical activity is evaluated with both self-reported and objective measurements [16]. More precisely, regarding subjective evaluation, the Tegner activity scale, Marx activity scale, International physical activity questionnaire, and Godin leisure time exercise questionnaire most frequently assessed the physical activity participation in this population. For instance, using the Tegner activity scale, the physical activity level of respondents who were subjected to the ACLR was 6.5, and no differences were recorded compared to the healthy controls [17]. Additionally, according to the Marx activity scale, the physical activity of the ACLR population markedly declined two years following surgery relative to the baseline values [18,19]. In terms of objective quantification of physical exercise, triaxial accelerometers, pedometers, and commercial devices like Fitbit Inc. were highlighted as commonly implemented measuring tools [16]. In particular, the literature reported that participants with a history of ACLR spent 147 min in moderate-to-vigorous physical activity per week [20] and 79 min in moderate-to-vigorous physical activity per day [21]. Finally, they also spent significantly less time engaging in moderate-to-vigorous physical exercise per week [20] and daily [21] compared to their noninjured counterparts.

The relationship between BMI and the level of physical activity in individuals without ACLR has already been broadly investigated [[22], [23], [24], [25], [26], [27]]. More specifically, the influence of BMI on physical activity engagement was explored in the general population, involving healthy elderly [25] and middle-aged adults [22,27], as well as in children [24]. In nearly all of the studies, the results obtained showed that increased values of BMI induced a reduction in physical activity participation. For example, an inverse association between BMI and subjectively assessed physical activity, which included time spent walking and engagement in sports activities, was documented in healthy middle-aged adults [27]. Similarly, higher total physical activity measured with accelerometers was correlated with lower values of BMI in men and women aged approximately 80 years [25]. As expected, in children, a negative relationship between BMI *z*-score and the level of physical activity has been revealed [24].

Indeed, numerous systematic reviews investigated the influence of BMI on self-reported outcomes, such as knee function [28,29] and quality of life [29,30], among respondents with a history of ACLR. On the other hand, the impact of BMI on the physical activity level, which also represents a patient-reported outcome, still needs to be comprehensively summarized. Hence, the objective of the presented research was to systematically examine the association between BMI and physical activity in individuals that were subjected to the surgery of the ACL. The authors hypothesized that the literature would report an inverse relationship between the examined variables. In other words, higher values of BMI will likely negatively influence the physical engagement of respondents.

## 2 Methods

### 2.1 Study design and protocol registration

The presented systematic literature review was conducted in line with each requirement and the guidelines provided in the Preferred Reporting Items for Systematic Review and Meta-Analyses (PRISMA) statement [31]. In addition, the PRISMA checklist is available online as supplementary material. The protocol for this investigation was registered in the International Prospective Register of Systematic Reviews (PROSPERO) with the following registration number: CRD42023457148.

### 2.2 Databases search and study selection

A comprehensive literature search was carried out via three electronic databases, including Web of Science, Scopus, and PubMed, from inception to September 2023. Of note, experts from the fields of sports science and medicine were additionally consulted to enhance the search strategy and ensure relevant keywords. It is indispensable to mention that a Boolean search syntax with operators "AND," "OR," and "NOT" has also been implemented. The example of the PubMed search and the keywords used are as follows: ("body mass index" OR "overweight" OR "obesity" OR "body fat") AND ("physical activity" OR "physical exercise" OR "exercise" OR "Tegner activity scale" OR "Marx activity scale") AND ("anterior cruciate ligament reconstruction" OR "anterior cruciate ligament injury" OR "anterior cruciate ligament surgery" OR "knee injuries"). Regarding the collection of additional evidence, reference citation lists and Google Scholar have been thoroughly searched. The study selection process implied screening of titles and abstracts of identified articles and analysis of investigations assessed for eligibility. Two reviewers (TT and RM) independently performed a search of databases and additional sources, as well as a selection of available literature, while potential disagreements were resolved via discussion until consensus was reached.

### 2.3 Eligibility criteria

The following inclusion criteria needed to be fulfilled: (1) study design was observational, such as cohort, case-control, and cross-sectional research; (2) participants underwent the surgery of the ACL, irrespective of reconstruction types. There were no restrictions concerning demographic variables of respondents, including age or gender; (3) BMI was evaluated as a predictor (independent) variable; and (4) assessed outcomes referred to the physical activity of the examined population.

Conversely, studies were excluded if: (1) in addition to the ACL rupture, other knee injuries were observed; (2) it was emphasized that the ACL injury was treated non-surgically; and (3) articles were not written in the English language. At last, case reports, conference papers, editorials, doctoral theses, not-peer-reviewed journal articles, systematic reviews and meta-analyses, and not-full-text available studies were also not deemed adequate for inclusion in this investigation.

### 2.4 Data extraction

Two independent reviewers (AB and AC) retrieved relevant data from all of the studies that satisfied the eligibility criteria and were involved in the qualitative analysis. Data extraction was conducted using a Microsoft Excel template. Firstly, data pertaining to the names of the authors, year of study publication, study design, and level of evidence were retrieved and presented in the manuscript. Secondly, demographic characteristics of the examined population, such as the number of included subjects, gender, and mean age, were also extracted with a Microsoft Excel template. Regarding surgical parameters, reconstruction type (primary or revision), follow-up period, and graft source were exhibited. It is necessary to emphasize that the follow-up period represents the time between ACLR and the evaluation of estimated outcomes. Thirdly, BMI values, instruments that assessed the physical activity of respondents, and the major findings of each research were also retrieved. Since all relevant data were provided in the analyzed investigations, the corresponding authors were not contacted via e-mail. Any inconsistencies among reviewers were solved after meeting and discussion. If a consensus has not been reached, the first investigator (SN) is consulted for clarification.

### 2.5 Assessment of risk of bias

National Institutes of Health (NIH) Quality Assessment Tool for Observational Cohort and Cross-Sectional Studies was applied for assessment of the risk of bias [32]. NIH encompasses 14 items that are related to the research objective, study population, a description of the exposure and the outcomes, a follow-up period, and statistical aspects of the investigations. There are five response options for each question, involving "yes," "no," "cannot be determined," "not applicable," and "not reported." Most importantly, the overall score of studies was "good," "fair," and "poor," indicating a "low risk of bias," "some concerns," and a "high risk of bias," respectively. In fact, articles were rated as "good" if "yes" was the response for between 9 and 14 items, indicating a "low risk of bias." Further, the methodological quality of studies was "fair" if a "yes" was a response for between 6 and 8 questions, indicating "some concerns." Investigations were rated as "poor" if "yes" was given to between 0 and 5 items, indicating a "high risk of bias" [33]. The evaluation of methodological quality was carried out by two reviewers (MM and RR). All discrepancies among them were resolved through discussion until an agreement was reached.

### 2.6 Data synthesis

Considering that a high level of methodological heterogeneity among studies has been observed, particularly in terms of measuring tools that assessed physical activity, a meta-analysis was not performed. Therefore, a qualitative synthesis approach has been applied. More precisely, all investigations were described in detail and presented in a tabular format.

## 3 Results

### 3.1 Literature search results

**Fig. 1:**
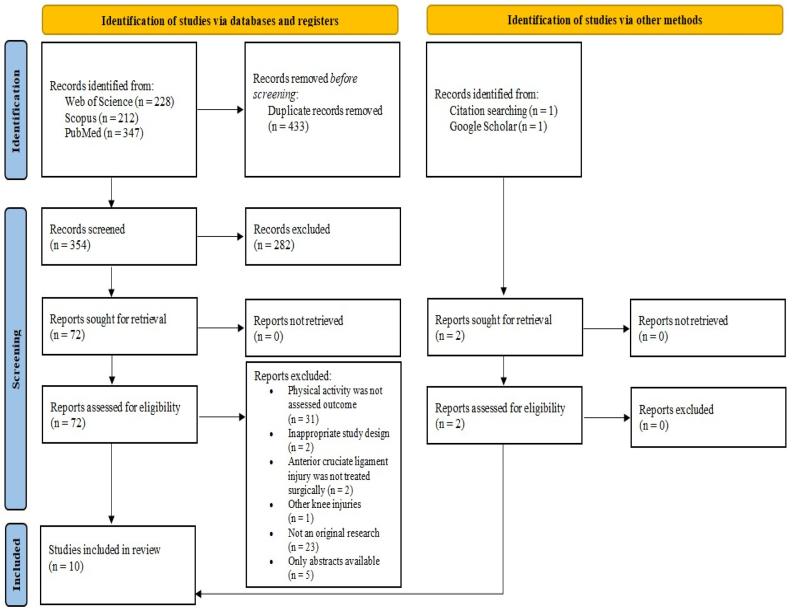
PRISMA flow diagram of the literature selection process.

A comprehensive search of the three databases yielded a total of 787 records. After removing 433 duplicates using Zotero software, the titles and abstracts of 354 reports were screened. Further, 72 full-text articles were assessed for eligibility following the elimination of 282 trials. Then, 64 studies did not fulfill the inclusion criteria, and a total of 8 reports were included in the final analysis. Additionally, two records have been identified via citation searching and a thorough search of Google Scholar. Overall, ten original research that examined the impact of BMI on physical activity levels in individuals with a history of ACLR were included in the qualitative analysis of this systematic review. Fig. 1 illustrates all aspects of the literature search and study selection process.

### 3.2 Characteristics of the included studies

All investigations were available in electronic databases between 2010 and 2021. Out of 10 observational studies, there were 7 cohort [[34], [35], [36], [37], [38], [39],^40^] and 3 cross-sectional [[41], [42], [43]] research. In terms of the level of evidence, one study reported level I [35], 5 investigations level II [36,38,39,42,40], and 4 articles level III [34,37,41,43]. Furthermore, 7171 individuals with a history of ACLR participated in studies that were involved in the final analysis. Four thousand eighty males and 3091 females were recorded, with a mean age of 25.5 years. Concerning reconstruction types, primary unilateral ACLR was most common, while revision surgery was also observed in several studies. A follow-up period was reported in nine [[34], [35], [36], [37], [38], [39],[40], [42], [43]] of ten research. The average time between ACLR and the evaluation of the influence of BMI on the physical activity of the examined population was approximately four years. Lastly, bone-tendon bone, hamstring autograft, Achilles tendon, and allografts were most frequently applied relating to the graft source. More details regarding study design, demographic variables, and surgical parameters are given in Table 1.

**Table 1:** Study design, demographic, and surgical parameters of the examined population.

Author (year)	Study design	Level of evidence	Sample size and gender	Mean age (years)	Reconstruction type	Follow-up period	Graft source
Bin Abd Razak et al., 2017 [34]	Retrospective cohort	III	118 male and 23 female participants	28.9 ± 5.9	Unilateral primary ACLR	2 years	Hamstring autograft
Cox et al., 2014 [35]	Multicenter cohort prognosis	I	785 male and 626 female respondents	23.1 ± 2.7	Unilateral primary and revision ACLR	6 years	BTB, hamstring autograft, tibialis anterior or posterior, and Achilles tendon
Dunn et al., 2010 [36]	Multicenter cohort	II	222 male and 171 female individuals	27.2 ± 11.1	Unilateral primary and revision ACLR	2 years	BTB, hamstring autograft, iliotibial band, quadriceps tendon, Achilles tendon, and allograft
Jones et al., 2019 [37]	Cohort	III	371 male and 304 female subjects	20.1 ± 2.6	Primary and revision ACLR	2 years	BTB autograft, hamstring autograft, and allograft
Jurkonis et al., 2018 [38]	Prospective cohort	II	159 male and 55 female patients	33.2 ± 9.8	Unilateral primary and revision ACLR	1 year	Hamstring tendon grafts
Moon Knee Group 2018 [39]	Longitudinal prospective cohort	II	902 male and 690 female participants	24.0 ± 2.7	Unilateral primary and revision ACLR	10 years	BTB autograft and allografts
Nguyen et al., 2017 [41]	Cross-sectional	III	662 male and 530 female respondents	23.1 ± 2.7	Unilateral primary ACLR	NA	NA
Pietrosimone et al., 2018 [42]	Cross-sectional prognostic	II	247 male and 421 female individuals	21.7 ± 6.2	Unilateral primary ACLR	2 years	Patellar tendon autograft, semitendinosus or gracilis autografts, and allograft
Ristic et al., 2021 [43]	Cross-sectional	III	413 male and 97 female subjects	27.0 ± 7.8	Primary and revision ACLR	3.5 years	NA
Spindler et al., 2011 [40]	Population cohort	II	201 male and 174 female patients	26.9 ± 11.2	Unilateral primary and revision ACLR	6 years	BTB, hamstring autograft, Achilles tendon, and allograft

Abbreviations: ACLR – anterior cruciate ligament reconstruction; BTB – bone-tendon-bone; NA – not applicable.

### 3.3 The impact of BMI on physical activity participation after ACLR

The mean BMI of respondents who underwent ACL surgery was 24.9 kg/m^2^. In each study, physical activity was evaluated subjectively using specific questionnaires. Specifically, the Tegner activity scale, which assesses and describes the level of work or physical activity of respondents, has been implemented in 4 studies [34,38,42,43]. Similarly, Marx activity scale, which evaluates various activity aspects of individuals with knee injuries, such as pivoting, running, cutting, and deceleration, was used in 6 papers [[35], [36], [37],^39^,^41^,^40^]. Most importantly, in 9 [[34], [35], [36], [37], [38], [39], [41], [42], [43]] out of 10 investigations, a statistically significant relationship between BMI and physical activity was revealed, irrespective of the questionnaire applied. Relating to the Tegner activity scale, an inverse correlation among variables was noted. Accordingly, increased values of BMI harmfully influenced the physical activity engagement of patients undergoing the ACLR. Likewise, in studies in which the Marx activity scale was implemented, lower BMI values were associated with higher levels of physical activity. Interestingly, it is crucial to highlight that two research [34,38] revealed that respondents in the groups with a BMI of 25 kg/m^2^ or more had noticeably reduced levels of physical activity participation relative to the individuals whose BMI was below 25 kg/m^2^ (Table 2).

**Table 2:** The influence of BMI on physical activity levels in individuals who underwent ACLR.

Author (year)	BMI (kg/m^2^)	Physical activity assessment	Main findings
Bin Abd Razak et al., 2017 [34]	25.5 ± 2.7	Tegner activity scale	The normal-BMI group (BMI <25 kg/m^2^) had a markedly higher Tegner score relative to the high-BMI group (BMI ≥25 kg/m^2^).
Cox et al., 2014 [35]	24.8 ± 0.8	Marx activity scale	Higher BMI negatively predicted the physical activity level of the examined population.
Dunn et al., 2010 [36]	25.5 ± 4.5	Marx activity scale	A statistically significant negative relationship between BMI values and physical activity participation was observed.
Jones et al., 2019 [37]	24.4 ± 0.9	Marx activity scale	The BMI of respondents negatively affected the level of their physical activity engagement.
Jurkonis et al., 2018 [38]	25.2 ± 3.9	Tegner activity scale	The normal BMI group (BMI <25 kg/m^2^) demonstrated a substantially higher Tegner score compared to the overweight group (BMI ≥25 kg/m^2^).
Moon Knee Group 2018 [39]	25.0 ± 0.8	Marx activity scale	An increased value of BMI predicted lower physical activity levels of the respondents.
Nguyen et al., 2017 [41]	25.0 ± 0.9	Marx activity scale	A strong inverse association between BMI and physical activity participation of patients was noted.
Pietrosimone et al., 2018 [42]	24.4 ± 3.7	Tegner activity scale	Higher values of BMI significantly correlated with lower Tegner activity levels among respondents.
Ristic et al., 2021 [43]	24.7 ± 3.0	Tegner activity scale	An increase in BMI was a factor that negatively impacted the level of physical activity.
Spindler et al., 2011 [40]	24.1 ± 4.4	Marx activity scale	No statistically significant relationship between BMI and physical activity engagement of patients has been revealed.

Abbreviations: ACLR – anterior cruciate ligament reconstruction; BMI – body mass index.

### 3.4 Assessment of risk of bias

Five studies [35,36,39,42,40] were rated as "good," indicating a "low risk of bias," while 4 [34,37,38,41] have been evaluated as "fair," indicating "some concerns." Only one article [43] had a "high risk of bias" due to the majority of responses being "no," "cannot be determined," "not reported," and "not applicable." In general, the lowest bias has been observed in aspects like research objective, items relating to the study population, sample size justification, and description of evaluated outcomes. In contrast, the main issue was noted in aspects such as assessment of exposure before measurements of the outcomes, repeated measure of exposure, and blinding of outcome assessors. The methodological quality evaluation of each study is available in Table 3.

**Table 3:** Risk of bias assessment of the included studies.

Author (year)	1	2	3	4	5	6	7	8	9	10	11	12	13	14	Overall score
Bin Abd Razak et al., 2017 [34]	Yes	Yes	Yes	Yes	Yes	No	Yes	Yes	CD	No	CD	NR	Yes	No	Fair
Cox et al., 2014 [35]	Yes	Yes	Yes	Yes	Yes	CD	Yes	No	Yes	No	CD	NR	Yes	Yes	Good
Dunn et al., 2010 [36]	Yes	Yes	Yes	Yes	NR	Yes	Yes	No	CD	No	Yes	NR	Yes	Yes	Good
Jones et al., 2019 [37]	Yes	Yes	Yes	No	NR	CD	Yes	No	Yes	CD	Yes	NR	Yes	CD	Fair
Jurkonis et al., 2018 [38]	Yes	Yes	Yes	Yes	CD	Yes	No	Yes	CD	No	Yes	NR	Yes	No	Fair
Moon Knee Group 2018 [39]	Yes	Yes	Yes	Yes	Yes	Yes	Yes	No	Yes	Yes	Yes	NR	Yes	Yes	Good
Nguyen et al., 2017 [41]	Yes	Yes	CD	Yes	CD	CD	NA	Yes	Yes	NA	Yes	NR	CD	Yes	Fair
Pietrosimone et al., 2018 [42]	Yes	Yes	CD	Yes	Yes	CD	Yes	Yes	Yes	No	Yes	NR	CD	Yes	Good
Ristic et al., 2021 [43]	Yes	Yes	No	No	CD	CD	NA	CD	Yes	NA	Yes	NR	CD	No	Poor
Spindler et al., 2011 [40]	Yes	Yes	Yes	CD	CD	Yes	Yes	Yes	Yes	No	Yes	NR	Yes	CD	Good

Abbreviations: CD – cannot determine; NA – not applicable; NR – not reported. 1 – research objective; 2 – study population; 3 – participation rate; 4 – respondents inclusion and exclusion criteria; 5 – sample size justification; 6 – exposure evaluated before outcome measurement; 7 – the length of the follow-up period; 8 – different levels of the exposure of interest; 9 – clarity of exposure measure; 10 – repeated exposure assessment; 11 – validity and reliability of the outcome measure; 12 – blinding of outcome assessors; 13 – follow-up rate was 80 % or more; 14 – analysis of confounders. Good – low risk of bias; fair – some concerns; poor – high risk of bias.

## 4 Discussion

### 4.1 Summary of evidence

The goal of this systematic literature review was to explore the influence of BMI on the level of physical activity in individuals with a history of ACLR. As hypothesized, the obtained results unambiguously demonstrated that BMI was inversely correlated with the physical activity of respondents. More precisely, higher values of BMI were identified as a factor that correlated with the diminished physical engagement of the analyzed population. Scientific literature found that a BMI over 25 kg/m^2^ most likely induced a decline in physical exercise in respondents undergoing ACLR [34,38]. Overall, overweight and obesity are very strong negative predictors of subjectively evaluated physical activity using the Tegner and Marx activity scale following ACL surgery.

### 4.2 Comparisons with the body of knowledge referring to the other self-reported outcomes

There is abundant scientific evidence regarding the relationship between BMI and the functionality of the knee joint following ACLR [[44], [45], [46], [47], [48], [49], [50], [51]]. The main findings of these investigations are consistent with the results obtained in the presented literature review. More specifically, an increased BMI predicted exacerbation of knee function among respondents who underwent the ACLR. For instance, higher values of BMI were significantly associated with lower Knee injury and Osteoarthritis Outcome Score subscales, such as pain and activities of daily living, as well as sport and recreation [45,46]. Likewise, BMI represented the factor that negatively predicted the International Knee Documentation Committee score one year following the surgery of the ACL [48]. In addition, BMI below 25 kg/m^2^ positively correlated with International Knee Documentation Committee 2000 score 6 and 12 months after ACLR [50]. Further, Griffith et al. [51] examined the relationship between BMI and knee function estimated with the Lysholm scale. Interestingly, the authors reported that participants with a BMI greater than 28 kg/m^2^ had considerably lower Lysholm scores compared to the individuals whose BMI was less than 28 kg/m^2^. In summary, as already highlighted , it is apparent that BMI represents a risk factor for decreased physical activity levels in the ACLR population. Therefore, body weight control is indispensable for the prevention of potentially impaired physical activity participation following ACLR.

The influence of BMI on quality of life has been extensively investigated [[52], [53], [54], [55], [56], [57], [58]]. There is quite compelling evidence that BMI is markedly correlated with the quality of life among individuals with a history of ACL surgery. More precisely, as in case with physical activity, the majority of the available literature unequivocally demonstrated a strong negative relationship between BMI and quality of life among respondents, including health-related quality of life, knee-related quality of life, and ACL-specific quality of life. For example, lower values of BMI were significant predictors of a higher physical component of health-related quality of life in a 6-year multicenter cohort research [54]. Analogously, an inverse association between BMI and the quality of life estimated with Knee injury and Osteoarthritis Outcome Score has also been documented [56]. Moreover, the impact of BMI on ACL-specific quality of life was explored in a sample of Australian participants who underwent ACLR [58]. The main findings indicated that increased BMI was negatively associated with quality of life. Additionally, the authors emphasized that higher values of BMI significantly correlated with lower health-related quality of life assessed with the Assessment of Quality of life-8D questionnaire. Overall, a firm body of evidence suggested that increased BMI was identified as a factor that detrimentally affected analyzed self-reported outcome, such as subjectively assessed physical activity engagement after surgery of ACL. Thus, stimulations of a healthy lifestiyle appears indispensable and crurial for the ACLR population.

### 4.3 Health, clinical, and practical implications of the results

As previously highlighted, a BMI over 25 kg/m^2^ elicited decreased physical activity levels in the examined population. Most importantly, the amount of physical exercise has been linked with countless health parameters [[59], [60], [61], [62], [63]]. Lower levels of physical activity were independently correlated with the higher values of various novel and traditional cardiovascular biomarkers [59]. Furthermore, physical inactivity represented a significant risk factor for all-cause mortality [60]. On the other hand, participation in regular physical exercise considerably reduced the risk of coronary heart disease, cardiovascular diseases, and stroke [62]. Similarly, regular physical exercise efficiency prevented the occurrence of numerous chronic diseases, such as diabetes, cancer, and obesity [61]. Lastly, it is noteworthy to emphasize that practicing physical activity was inversely associated with mental health issues among adults in the United States [63]. Therefore, taking into account that increased values of BMI reduce engagement in physical activity in individuals with a history of ACLR, the implementation of different lifestyle interventions appears necessary to prevent the exacerbation of previously specified health variables related to the level of physical activity. Specifically, a combination of appropriately designed physical exercise interventions and optimal nutrition is considered crucial for the enhancement of the health status of this population. In other words, the application of aerobic and strength training modalities with a balanced consumption of carbohydrates, fats, and proteins should lead to a reduction in body weight in overweight and obese individuals who were subjected to the ACLR. Finally, cooperation between sports and medical scientists, as well as exercise specialists and medical practitioners, would be very useful regarding the correction and control of BMI values and consequently improvements in physical activity participation that probably will prevent impairments in the already mentioned health parameters.

### 4.4 Strengths, limitations, and gaps in the literature

There are several key strengths of the presented systematic review that are indispensable to emphasize. Both genders were almost equally involved in all investigations. Hence, the results obtained can be generalized to the males and females who underwent ACLR. Seven out of 10 included studies have been published within the last 5 years; namely, the findings synthesized in this research are quite innovative and actual. In terms of reconstruction types, both primary and revision surgery of ACL were implemented in the majority of available literature. However, the following examinations should focus on the influence of BMI on physical activity participation in a sample of respondents that underwent exclusively the revision or bilateral surgery of ACL.

Conversely, certain obvious limitations must be taken into account during the interpretation of the obtained results. In each of the studies, physical activity was evaluated subjectively using Tegner and Marx activity scales. Thus, future studies investigating the relationship between BMI and objectively quantified physical activity are warranted. Further, concerning the methodological quality, "some concerns" and "high risk of bias" were very common among investigations included in the qualitative analysis. Additionally, considering that the average time between the surgery and assessments of relevant outcomes was approximately 4 years, studies with long-term follow-up periods, including 10 years or more, are extremely needed. Concerning demographic variables, the age range of the examined population was between 21 and 33 years. Therefore, it is highly recommended in the future to evaluate the impact of BMI on the physical activity of middle-aged and particularly older adults who were subjected to the ACLR.

## 5 Conclusion

The main findings of this study unequivocally demonstrated that increased BMI was associated with reduced physical activity participation in individuals with a history of ACL surgery. Moreover, based on the available scientific evidence, it is apparent that BMI over 25 kg/m^2^ negatively affected physical engagement following ACLR. Thus, taking into account the health implications of diminished levels of physical activity, various lifestyle interventions involving a combination of adequately created physical exercise programs and optimal nutrition are highly desirable. In other words, sports and medical scientists, as well as exercise specialists and medical practitioners, need to stimulate a healthy lifestyle, particularly in overweight and obese individuals undergoing the ACLR, to prevent exacerbation of previously highlighted health parameters. Finally, studies exploring the relationship between BMI and objectively measured physical activity after ACLR are warranted.

## Funding

The presented investigation was funded by the Provincial Secretariat for Higher Education and Scientific Research, grant number 142-451-3098.

## Data availability statement

All relevant data are included in the article and the supplementary material.

## Additional information

The PRISMA checklist is provided as supplementary material.

## CRediT authorship contribution statement

**Srdjan Ninkovic:** Writing – review & editing, Writing – original draft, Methodology, Data curation, Conceptualization. **Marko Manojlovic:** Writing – review & editing, Writing – original draft, Methodology, Data curation, Conceptualization. **Roberto Roklicer:** Writing – review & editing, Writing – original draft, Methodology. **Antonino Bianco:** Writing – review & editing, Writing – original draft, Methodology. **Attilio Carraro:** Writing – review & editing, Writing – original draft, Methodology. **Radenko Matic:** Writing – review & editing, Writing – original draft. **Tatjana Trivic:** Writing – review & editing, Writing – original draft, Methodology. **Patrik Drid:** Writing – review & editing, Writing – original draft, Methodology, Formal analysis, Conceptualization.

## Declaration of competing interest

The authors declare that they have no known competing financial interests or personal relationships that could have appeared to influence the work reported in this paper.

## References

[1] Sanders T.L., Maradit Kremers H., Bryan A.J., Larson D.R., Dahm D.L., Levy B.A., Stuart M.J., Krych A.J. (2016). Incidence of anterior cruciate ligament tears and reconstruction: a 21-year population-based study. Am. J. Sports Med..

[2] Paudel Y.R., Sommerfeldt M., Voaklander D. (2023). Increasing incidence of anterior cruciate ligament reconstruction: a 17-year population-based study. Knee Surg. Sports Traumatol. Arthrosc..

[3] Vaishya R., Agarwal A.K., Ingole S., Vijay V. (2015). Current trends in anterior cruciate ligament reconstruction: a review. Cureus.

[4] Kim H.S., Seon J.K., Jo A.R. (2013). Current trends in anterior cruciate ligament reconstruction. Knee Surg. Relat. Res..

[5] Herzog M.M., Marshall S.W., Lund J.L., Pate V., Mack C.D., Spang J.T. (2017). Incidence of anterior cruciate ligament reconstruction among adolescent females in the United States, 2002 through 2014. JAMA Pediatr..

[6] Mall N.A., Chalmers P.N., Moric M., Tanaka M.J., Cole B.J., Bach B.R., Paletta G.A. (2014). Incidence and trends of anterior cruciate ligament reconstruction in the United States. Am. J. Sports Med..

[7] Buller L.T., Best M.J., Baraga M.G., Kaplan L.D. (2014). Trends in anterior cruciate ligament reconstruction in the United States, Orthop. J. Sports Med..

[8] Norberg C., Hallström Stalin U., Matsson L., Thorngren-Jerneck K., Klingberg G. (2012). Body mass index (BMI) and dental caries in 5-year-old children from southern Sweden, Community Dent. Oral Epidemiol.

[9] WHO (1995). https://iris.who.int/handle/10665/37003.

[10] Nuttall F.Q. (2015). Body mass index: obesity, BMI, and health: a critical review. Nutr. Today.

[11] Evans B., Colls R. (2009). Measuring fatness, governing bodies: the spatialities of the Body mass index (BMI) in anti‐obesity politics. Antipode.

[12] Dixon J.B. (2010). The effect of obesity on health outcomes. Mol. Cell. Endocrinol..

[13] Ucan O., Ovayolu N. (2010). Relationship between diabetes mellitus, hypertension and obesity, and health-related quality of life in Gaziantep, a central south-eastern city in Turkey. J. Clin. Nurs..

[14] Busutil R., Espallardo O., Torres A., Martínez-Galdeano L., Zozaya N., Hidalgo-Vega Á. (2017). The impact of obesity on health-related quality of life in Spain. Health Qual. Life Outcome.

[15] Prickett C., Brennan L., Stolwyk R. (2015). Examining the relationship between obesity and cognitive function: a systematic literature review. Obes. Res. Clin. Pract..

[16] Kuenze C., Collins K., Pfeiffer K.A., Lisee C. (2022). Assessing physical activity after ACL injury: moving beyond return to sport. Sport Health.

[17] Triplett A.N., Kuenze C.M. (2021). Characterizing body composition, cardiorespiratory fitness, and physical activity in women with anterior cruciate ligament reconstruction. Phys. Ther. Sport.

[18] Antosh I.J., Svoboda S.J., Peck K.Y., Garcia E.J., Cameron K.L. (2018). Change in KOOS and WOMAC Scores in a young athletic population with and without anterior cruciate ligament injury. Am. J. Sports Med..

[19] Nwachukwu B.U., Voleti P.B., Chang B., Berkanish P., Mahony G.T., Williams R.J., Altchek D.W., Allen A.A. (2017). Comparative influence of sport type on outcome after anterior cruciate ligament reconstruction at minimum 2-year follow-up. Arthrosc. J. Arthrosc. Relat. Surg..

[20] Kuenze C., Lisee C., Pfeiffer K.A., Cadmus-Bertram L., Post E.G., Biese K., Bell D.R. (2019). Sex differences in physical activity engagement after ACL reconstruction. Phys. Ther. Sport.

[21] Bell D.R., Pfeiffer K.A., Cadmus-Bertram L.A., Trigsted S.M., Kelly A., Post E.G., Hart J.M., Cook D.B., Dunn W.R., Kuenze C. (2017). Objectively measured physical activity in patients after anterior cruciate ligament reconstruction. Am. J. Sports Med..

[22] Van Dyck D., Cerin E., De Bourdeaudhuij I., Hinckson E., Reis R.S., Davey R., Sarmiento O.L., Mitas J., Troelsen J., MacFarlane D., Salvo D., Aguinaga-Ontoso I., Owen N., Cain K.L., Sallis J.F. (2015). International study of objectively measured physical activity and sedentary time with body mass index and obesity: IPEN adult study. Int. J. Obes..

[23] Schröder H., Marrugat J., Elosua R., Covas M.I. (2003). Relationship between body mass index, serum cholesterol, leisure-time physical activity, and diet in a Mediterranean Southern-Europe population. Br. J. Nutr..

[24] Remmers T., Sleddens E.F., Gubbels J.S., de Vries S.I., Mommers M., Penders J., Kremers S.P., Thijs C. (2014). Relationship between physical activity and the development of body mass index in children. Med. Sci. Sports Exerc..

[25] Bann D., Hire D., Manini T., Cooper R., Botoseneanu A., McDermott M.M., Pahor M., Glynn N.W., Fielding R., King A.C., Church T., Ambrosius W.T., Gill T.T. (2015). For the LIFE study group, Light intensity physical activity and sedentary behavior in relation to body mass index and grip strength in older adults: cross-sectional findings from the lifestyle interventions and independence for elders (LIFE) study. PLoS One.

[26] Gaylis J.B., Levy S.S., Hong M.Y. (2020). Relationships between body weight perception, body mass index, physical activity, and food choices in Southern California male and female adolescents. Int. J. Adolesc. Youth.

[27] Stamatakis E., Hirani V., Rennie K. (2009). Moderate-to-vigorous physical activity and sedentary behaviours in relation to body mass index-defined and waist circumference-defined obesity. Br. J. Nutr..

[28] DiSilvestro K.J., Jauregui J.J., Glazier E., Cherkalin D., Bennett C.H., Packer J.D., Henn R.F. (2019). Outcomes of anterior cruciate ligament reconstruction in obese and overweight patients: a systematic review. Clin. J. Sport Med..

[29] An V.V., Scholes C., Mhaskar V.A., Hadden W.J., Parker D. (2017). Limitations in predicting outcome following primary ACL reconstruction with single-bundle hamstring autograft - a systematic review. Knee.

[30] de Valk E.J., Moen M.H., Winters M., Bakker E.W., Tamminga R., van der Hoeven H. (2013). Preoperative patient and injury factors of successful rehabilitation after anterior cruciate ligament reconstruction with single-bundle techniques. Arthrosc. J. Arthrosc. Relat. Surg..

[31] Page M.J., McKenzie J.E., Bossuyt P.M., Boutron I., Hoffmann T.C., Mulrow C.D., Shamseer L., Tetzlaff J.M., Akl E.A., Brennan S.E., Chou R., Glanville J., Grimshaw J.M., Hróbjartsson A., Lalu M.M., Li T., Loder E.W., Mayo-Wilson E., McDonald S., McGuinness L.A., Stewart L.A., Thomas J., Tricco A.C., Welch V.A., Whiting P., Moher D. (2021). The PRISMA 2020 statement: an updated guideline for reporting systematic reviews. Int. J. Surg..

[32] National Heart L., Blood I. (2019). https://www.nhlbinih.gov/health-topics/study-quality-assessment-tools.

[33] Robinson K., Riley N., Owen K., Drew R., Mavilidi M.F., Hillman C.H., Faigenbaum A.D., Garcia-Hermoso A., Lubans D.R. (2023). Effects of resistance training on academic outcomes in school-aged youth: a systematic review and meta-analysis. Sports Med..

[34] Bin Abd Razak H.R., Chong H.C., Tan H.A. (2017). Obesity is associated with poorer range of motion and Tegner scores following hamstring autograft anterior cruciate ligament reconstruction in Asians. Ann. Transl. Med..

[35] Cox C.L., Huston L.J., Dunn W.R., Reinke E.K., Nwosu S.K., Parker R.D., Wright R.W., Kaeding C.C., Marx R.G., Amendola A., McCarty E.C., Spindler K.P. (2014). Are articular cartilage lesions and meniscus tears predictive of IKDC, KOOS, and Marx activity level outcomes after anterior cruciate ligament reconstruction? A 6-year multicenter cohort study. Am. J. Sports Med..

[36] Dunn W.R., Spindler K.P., the MOON Consortium (2010). Predictors of activity level 2 years after anterior cruciate ligament reconstruction (ACLR): a multicenter orthopaedic outcomes network (MOON) ACLR cohort study. Am. J. Sports Med..

[37] Jones M.H., Reinke E.K., Zajichek A., Kelley-Moore J.A., Khair M.M., Malcolm T.L., Moon Knee Group, Spindler K.P., Amendola A., Andrish J.T., Brophy R.H., Flanigan D.C., Huston L.J., Kaeding C.C., Marx R.G., Matava M.J., Parker R.D., Wolf B.R., Wright R.W. (2019). Neighborhood socioeconomic status affects patient-reported outcome 2 years after ACL reconstruction. Orthop. J. Sports Med..

[38] Jurkonis R., Gudas R., Smailys A. (2018). Influence of graft diameter on functional outcomes after anterior cruciate ligament reconstruction: a prospective study with a 1-year follow-up. Med. Sci. Mon. Int. Med. J. Exp. Clin. Res..

[39] Spindler K.P., Huston L.J., Chagin K.M., Kattan M.W., Reinke E.K., Amendola A., Andrish J.T., Brophy R.H., Cox C.L., Dunn W.R., Flanigan D.C., Jones M.H., Kaeding C.C., Magnussen R.A., Marx R.G., Matava M.J., McCarty E.C., Parker R.D., Pedroza A.D., Vidal A.F., Wolcott M.L., Wolf B.R., Wright R.W., MOON Knee Group (2018). Ten-year outcomes and risk factors after anterior cruciate ligament reconstruction: a MOON longitudinal prospective cohort study. Am. J. Sports Med..

[40] Spindler K.P., Huston L.J., Wright R.W., Kaeding C.C., Marx R.G., Amendola A., Parker R.D., Andrish J.T., Reinke E.K., Harrell F.E., Moon Group, Dunn W.R. (2011). The prognosis and predictors of sports function and activity at minimum 6 years after anterior cruciate ligament reconstruction: a population cohort study. Am. J. Sports Med..

[41] Nguyen J.T., Wasserstein D., Reinke E.K., Spindler K.P., Mehta N., Doyle J.B., Moon Group, Marx R.G. (2017). Does the chronicity of anterior cruciate ligament ruptures influence patient-reported outcomes before surgery?. Am. J. Sports Med..

[42] Pietrosimone B., Kuenze C., Hart J.M., Thigpen C., Lepley A.S., Blackburn J.T., Padua D.A., Grindstaff T., Davis H., Bell D. (2018). Weak associations between body mass index and self-reported disability in people with unilateral anterior cruciate ligament reconstruction. Knee Surg. Sports Traumatol. Arthrosc..

[43] Ristić V., Šumar V., Rašović P., Milankov V. (2021). The impact of body mass index on the results of anterior cruciate ligament reconstruction. Med. Pregl..

[44] Wasserstein D., Huston L.J., Nwosu S., Moon Group, Kaeding C.C., Parker R.D., Wright R.W., Andrish J.T., Marx R.G., Amendola A., Wolf B.R., McCarty E.C., Wolcott M., Dunn W.R., Spindler K.P. (2015). KOOS pain as a marker for significant knee pain two and six years after primary ACL reconstruction: a multicenter orthopaedic outcomes network (MOON) prospective longitudinal cohort study. Osteoarthritis Cartilage.

[45] Hamrin Senorski E., Svantesson E., Spindler K.P., Alentorn-Geli E., Sundemo D., Westin O., Karlsson J., Samuelsson K. (2018). Ten-year risk factors for inferior knee injury and osteoarthritis outcome score after anterior cruciate ligament reconstruction: a study of 874 patients from the Swedish national knee ligament register. Am. J. Sports Med..

[46] Sasaki S., Tsuda E., Hiraga Y., Yamamoto Y., Maeda S., Sasaki E., Ishibashi Y. (2016). Prospective randomized study of objective and subjective clinical results between double-bundle and single-bundle anterior cruciate ligament reconstruction. Am. J. Sports Med..

[47] Kowalchuk D.A., Harner C.D., Fu F.H., Irrgang J.J. (2009). Prediction of patient-reported outcome after single-bundle anterior cruciate ligament reconstruction. Arthrosc. J. Arthrosc. Relat. Surg..

[48] Patterson B.E., Culvenor A.G., Barton C.J., Guermazi A., Stefanik J.J., Crossley K.M. (2020). Patient-reported outcomes one to five years after anterior cruciate ligament reconstruction: the effect of combined injury and associations with osteoarthritis features defined on magnetic resonance imaging. Arthritis Care Res..

[49] MARS Group (2019). Relationship between sports participation after revision anterior cruciate ligament reconstruction and 2-year patient-reported outcome measures. Am. J. Sports Med..

[50] Magnitskaya N., Mouton C., Gokeler A., Nuehrenboerger C., Pape D., Seil R. (2020). Younger age and hamstring tendon graft are associated with higher IKDC 2000 and KOOS scores during the first year after ACL reconstruction. Knee Surg. Sports Traumatol. Arthrosc..

[51] Griffith T.B., Allen B.J., Levy B.A., Stuart M.J., Dahm D.L. (2013). Outcomes of repeat revision anterior cruciate ligament reconstruction. Am. J. Sports Med..

[52] Bernholt D.L., Dornan G.J., DePhillipo N.N., Aman Z.S., Kennedy M.I., LaPrade R.F. (2020). High-grade posterolateral tibial plateau impaction fractures in the setting of a primary anterior cruciate ligament tear are correlated with an increased preoperative pivot shift and inferior postoperative outcomes after anterior cruciate ligament reconstruction. Am. J. Sports Med..

[53] Nwachukwu B.U., Chang B., Voleti P.B., Berkanish P., Cohn M.R., Altchek D.W., Allen A.A., Williams Rd R.J. (2017). Preoperative short form health survey score is predictive of return to play and minimal clinically important difference at a minimum 2-year follow-up after anterior cruciate ligament reconstruction. Am. J. Sports Med..

[54] Dunn W.R., Wolf B.R., Harrell F.E., Reinke E.K., Huston L.J., Moon Knee Group, Spindler K.P. (2015). Baseline predictors of health-related quality of life after anterior cruciate ligament reconstruction: a longitudinal analysis of a multicenter cohort at two and six years. J. Bone Joint Surg..

[55] Galea-O'Neill R.J., Bruder A.M., Goulis J., Shields N. (2021). Modifiable factors and their association with self-reported knee function and activity after anterior cruciate ligament reconstruction: a systematic review and meta-analysis. Physiother. Theory Pract..

[56] Heijne A., Ang B.O., Werner S. (2009). Predictive factors for 12-month outcome after anterior cruciate ligament reconstruction. Scand. J. Med. Sci. Sports.

[57] Frobell R.B., Svensson E., Göthrick M., Roos E.M. (2008). Self-reported activity level and knee function in amateur football players: the influence of age, gender, history of knee injury and level of competition. Knee Surg. Sports Traumatol. Arthrosc..

[58] Filbay S.R., Ackerman I.N., Russell T.G., Crossley K.M. (2017). Return to sport matters-longer-term quality of life after ACL reconstruction in people with knee difficulties. Scand. J. Med. Sci. Sports.

[59] Mora S., Lee I.M., Buring J.E., Ridker P.M. (2006). Association of physical activity and body mass index with novel and traditional cardiovascular biomarkers in women. JAMA.

[60] Crespo C.J., Palmieri M.R., Perdomo R.P., Mcgee D.L., Smit E., Sempos C.T., Lee I.M., Sorlie P.D. (2002). The relationship of physical activity and body weight with all-cause mortality: results from the Puerto Rico Heart Health Program. Ann. Epidemiol..

[61] Warburton D.E., Nicol C.W., Bredin S.S. (2006). Health benefits of physical activity: the evidence. Can. Med. Assoc. J..

[62] Blair S.N., Cheng Y., Holder J.S. (2001). Is physical activity or physical fitness more important in defining health benefits?. Med. Sci. Sports Exerc..

[63] Chekroud S.R., Gueorguieva R., Zheutlin A.B., Paulus M., Krumholz H.M., Krystal J.H., Chekroud A.M. (2018). Association between physical exercise and mental health in 1·2 million individuals in the USA between 2011 and 2015: a cross-sectional study. Lancet Psychiatr..

